# Supplementary material for: Impact of the coronavirus disease 2019 pandemic on the diversity of notifiable infectious diseases: a case study in Shanghai, China
Source: PeerJ. 2024 Mar 12;12:e17124. doi: 10.7717/peerj.17124 (PMC10941765; doi:10.7717/peerj.17124)
Supplement: Supplemental Information 1 [file peerj-12-17124-s001.docx]

**Impact of the coronavirus disease 2019 pandemic on the diversity of notifiable infectious diseases: A case study in Shanghai, China**

Yongfang Zhang* and Wenli Feng*

School of Chemistry and Chemical Engineering, Zhoukou Normal University, Zhoukou, Henan, China

Corresponding Author:

Wenli Feng

No.6, Middle Wenchang Avenue, Zhoukou, Henan Province, 466001, China

Email address: wlfeng@zknu.edu.cn

**Section 1. Calculation of weight using the entropy weight method**

As shown in Table S1, our data is a matrix of i rows (48), j columns (2).

a. Data standardization using the method of negative indicators

$X_{ij}=\frac{X_{j max}-X_{ij}}{X_{j max}-X_{j min}}$ （1）

b. the weight of indictor j, and month i.

$$Y_{ij}=\frac{X_{ij}}{\sum_{i=1}^{m} X_{ij}} (2)$$

c. Calculate the information entropy of indicator.

$$e_{j}=-k\sum_{i=1}^{m} {(Y}_{ij}lnY_{ij}) (3)$$

d. Redundancy rate of the information entropy

$$d_{j}=1-e_{j} (4)$$

e. The weight of indicators

$$W_{i}=\frac{d_{j}}{\sum_{i=1}^{m} d_{j}} (5)$$

**Table S1.** The coefficients of weight were derived by using entropy method.

| **Number of cases** | **Types of diseases** |  | **X_ij_** | |  | **Y_ij_** | |  | **e_j_** | |
| --- | --- | --- | --- | --- | --- | --- | --- | --- | --- | --- |
| 10350 | 30 |  | 0.763 | 0.429 |  | 0.023 | 0.025 |  | 0.99366 | 0.99393 |
| 8332 | 30 |  | 0.826 | 0.429 |  | 0.025 | 0.025 |  |  |  |
| 10199 | 30 |  | 0.767 | 0.429 |  | 0.023 | 0.025 |  | **d_j_** |  |
| 9193 | 30 |  | 0.799 | 0.429 |  | 0.024 | 0.025 |  | 0.00634 | 0.00607 |
| 11071 | 31 |  | 0.740 | 0.286 |  | 0.023 | 0.017 |  | **W_i_** |  |
| 13060 | 31 |  | 0.677 | 0.286 |  | 0.021 | 0.017 |  | 0.511 | 0.489 |
| 13875 | 32 |  | 0.651 | 0.143 |  | 0.020 | 0.008 |  |  |  |
| 12884 | 32 |  | 0.682 | 0.143 |  | 0.021 | 0.008 |  |  |  |
| 13392 | 31 |  | 0.666 | 0.286 |  | 0.020 | 0.017 |  |  |  |
| 15138 | 31 |  | 0.611 | 0.286 |  | 0.019 | 0.017 |  |  |  |
| 13682 | 30 |  | 0.657 | 0.429 |  | 0.020 | 0.025 |  |  |  |
| 14795 | 31 |  | 0.622 | 0.286 |  | 0.019 | 0.017 |  |  |  |
| 11429 | 30 |  | 0.728 | 0.429 |  | 0.022 | 0.025 |  |  |  |
| 7351 | 31 |  | 0.857 | 0.286 |  | 0.026 | 0.017 |  |  |  |
| 8338 | 30 |  | 0.826 | 0.429 |  | 0.025 | 0.025 |  |  |  |
| 9557 | 33 |  | 0.788 | 0.000 |  | 0.024 | 0.010 |  |  |  |
| 16006 | 30 |  | 0.584 | 0.429 |  | 0.018 | 0.025 |  |  |  |
| 25245 | 32 |  | 0.292 | 0.143 |  | 0.009 | 0.008 |  |  |  |
| 28190 | 31 |  | 0.198 | 0.286 |  | 0.006 | 0.017 |  |  |  |
| 21461 | 32 |  | 0.411 | 0.143 |  | 0.013 | 0.008 |  |  |  |
| 26105 | 32 |  | 0.264 | 0.143 |  | 0.008 | 0.008 |  |  |  |
| 15334 | 31 |  | 0.605 | 0.286 |  | 0.019 | 0.017 |  |  |  |
| 10722 | 33 |  | 0.751 | 0.000 |  | 0.023 | 0.010 |  |  |  |
| 11332 | 30 |  | 0.732 | 0.429 |  | 0.022 | 0.025 |  |  |  |
| 19876 | 30 |  | 0.461 | 0.429 |  | 0.014 | 0.025 |  |  |  |
| 15200 | 33 |  | 0.609 | 0.000 |  | 0.019 | 0.010 |  |  |  |
| 16687 | 31 |  | 0.562 | 0.286 |  | 0.017 | 0.017 |  |  |  |
| 13320 | 32 |  | 0.669 | 0.143 |  | 0.020 | 0.008 |  |  |  |
| 13612 | 31 |  | 0.659 | 0.286 |  | 0.020 | 0.017 |  |  |  |
| 14142 | 31 |  | 0.643 | 0.286 |  | 0.020 | 0.017 |  |  |  |
| 14908 | 32 |  | 0.618 | 0.143 |  | 0.019 | 0.008 |  |  |  |
| 12482 | 31 |  | 0.695 | 0.286 |  | 0.021 | 0.017 |  |  |  |
| 15195 | 33 |  | 0.609 | 0.000 |  | 0.019 | 0.010 |  |  |  |
| 12048 | 29 |  | 0.709 | 0.571 |  | 0.022 | 0.034 |  |  |  |
| 10603 | 30 |  | 0.755 | 0.429 |  | 0.023 | 0.025 |  |  |  |
| 31663 | 32 |  | 0.089 | 0.143 |  | 0.003 | 0.008 |  |  |  |
| 34466 | 29 |  | 0.000 | 0.571 |  | 0.010 | 0.034 |  |  |  |
| 3460 | 29 |  | 0.980 | 0.571 |  | 0.030 | 0.034 |  |  |  |
| 2842 | 26 |  | 1.000 | 1.000 |  | 0.031 | 0.059 |  |  |  |
| 3629 | 29 |  | 0.975 | 0.571 |  | 0.030 | 0.034 |  |  |  |
| 3767 | 31 |  | 0.971 | 0.286 |  | 0.030 | 0.017 |  |  |  |
| 4613 | 29 |  | 0.944 | 0.571 |  | 0.029 | 0.034 |  |  |  |
| 5079 | 30 |  | 0.929 | 0.429 |  | 0.028 | 0.025 |  |  |  |
| 5638 | 31 |  | 0.912 | 0.286 |  | 0.028 | 0.017 |  |  |  |
| 7684 | 30 |  | 0.847 | 0.429 |  | 0.026 | 0.025 |  |  |  |
| 7963 | 29 |  | 0.838 | 0.571 |  | 0.026 | 0.034 |  |  |  |
| 7418 | 27 |  | 0.855 | 0.857 |  | 0.026 | 0.051 |  |  |  |
| 7463 | 27 |  | 0.854 | 0.857 |  | 0.026 | 0.051 |  |  |  |

**Fig. S1.** Monthly changes in meteorological factors (a) & (b), air pollutants (c) and socioeconomic indicators (d) from 2017 to 2020 in Shanghai (NASA, 2020; SBS, 2020); (e) Change in monthly confirmed cases of COVID-19 in the 2020 in Shanghai (SMHC, 2020).

**(a)**

**Fig.S2.** The comparison of the predicted values with the observed values.

**Table S2.** Parameters of the results predicted from the Optimized model. The forecasting performance was assessed using error metrics (*Hyndman & Athanasopoulos, 2018*) including mean absolute deviation (MAD), mean absolute percentage error (MAPE), and root mean square error (RMSE).

| **Parameter type** | **Parameter** | **Cases** | **Types** | **NDOI** |
| --- | --- | --- | --- | --- |
| Optimized model |  | Autoregression | Autoregression | Season superposing |
| Data statistics | Min | 7,351 | 29 | 0.396 |
|  | Mean | 14,633 | 31 | 0.568 |
|  | Max | 31,663 | 33 | 0.896 |
|  | Std. | 5,641 | 1.05 | 0.126 |
|  | Ljung-Box | 16 | 16.94 | 18.95 |
| Forecast accuracy | RMSE | 2,756 | 0.7 | 0.142 |
|  | MAD | 2,060 | 0.52 | 0.118 |
|  | MAPE | 14.08% | 1.69% | 20.17% |
|  | Durbin-Watson | 2.16 | 2.16 | 1.84 |
|  | Theil‘s U | — | — | 0.836 |
| Regression | Std. | 2,836 | 0.72 | — |
|  | R^2^ | 0.75 | 0.5435 | — |
|  | Adjust-R^2^ | 0.75 | 0.5301 | — |
| Smooth factor | Alpha | — | — | 0.139 |
|  | Gamma | — | — | 0.001 |


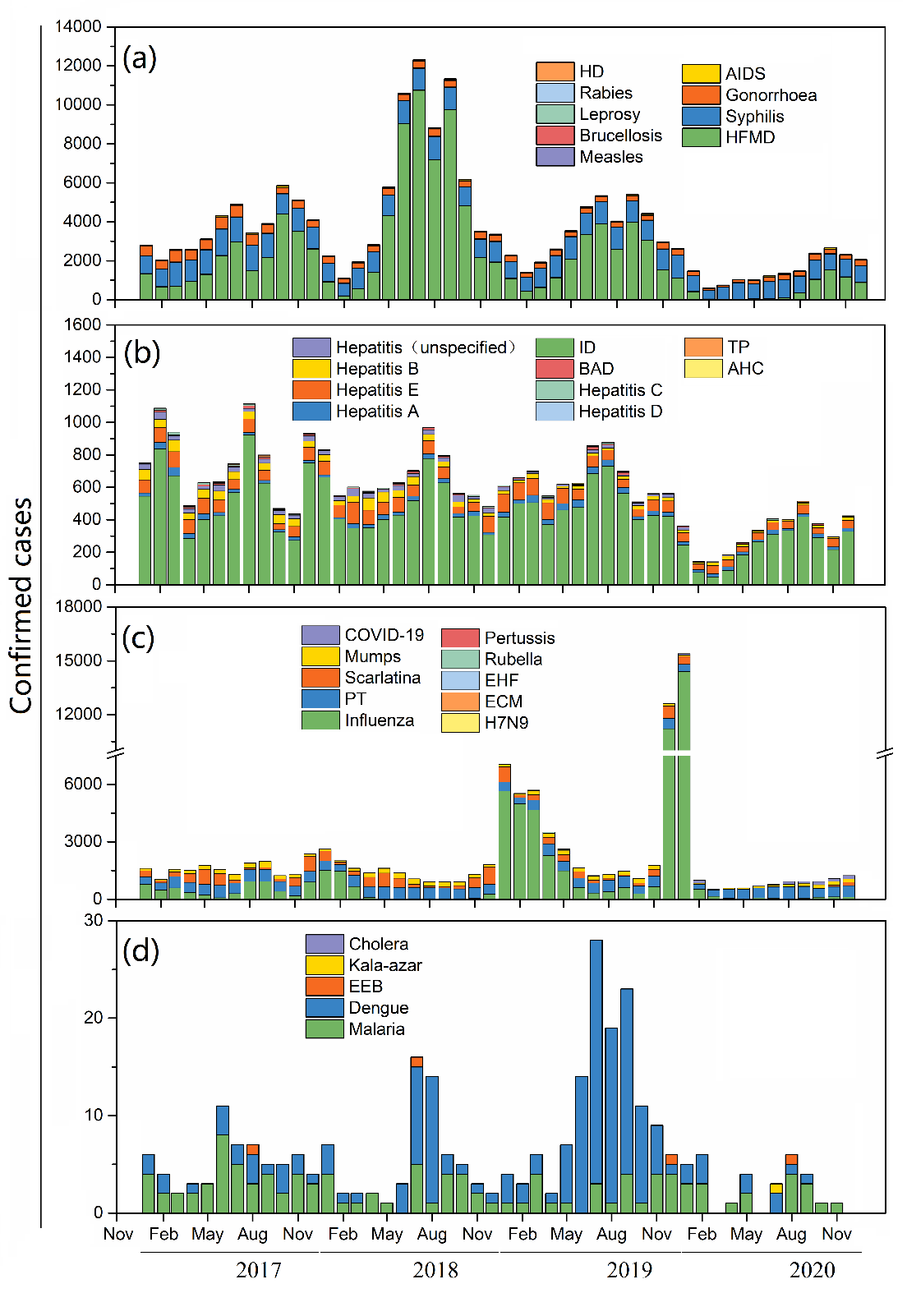


**Fig. S3.** Monthly changes in number of confirmed cases of notifiable infectious diseases transmitted by (a) direct-contact, (b) water and food, (c) airborne, (d) vector-borne

**Table S3.** Summary of DCA and RDA.

| DCA | Statistic | Axis 1 | Axis 2 | Axis 3 | Axis 4 |
| --- | --- | --- | --- | --- | --- |
|  | Eigenvalues | 0.0217 | 0.0107 | 0.0078 | 0.0043 |
|  | Explained variation (cumulative) | 27.92 | 41.71 | 51.79 | 57.38 |
|  | Gradient length | 0.5 | 0.48 | 0.48 | 0.37 |
| RDA | Statistic | Axis 1 | Axis 2 | Axis 3 | Axis 4 |
|  | Eigenvalues | 0.4868 | 0.2104 | 0.1203 | 0.0615 |
|  | Explained variation (cumulative) | 48.68 | 69.72 | 81.75 | 87.90 |
|  | Pseudo-canonical correlation | 0.973 | 0.8881 | 0.8306 | 0.8593 |
|  | Explained fitted variation (cumulative) | 53.21 | 78.02 | 81.16 | 90.36 |
|  | Permutation Test Results: |  |  |  |  |
|  | On All Axes | pseudo-F=6.5,  P=0.002 | |  |  |

**Table S4.** Correlation analysis.

|  | AP | TE | RH | WS | Prec. | AQI | PM_2.5_ | PM_10_ | SO_2_ | NO_2_ | CO | O_3_ | IT | SC | IO | APT | FV |
| --- | --- | --- | --- | --- | --- | --- | --- | --- | --- | --- | --- | --- | --- | --- | --- | --- | --- |
| **Direct-contact transmitted diseases** |  |  |  |  |  |  |  |  |  |  |  |  |  |  |  |  |  |
| Hand-foot-and-mouth disease | -0.401****** | 0.484****** | -0.134 | 0.044 | 0.075 | -0.154 | -0.428****** | -0.322***** | -0.062 | -0.366***** | -0.399****** | 0.146 | 0.439****** | -0.196 | 0.141 | 0.432****** | -0.164 |
| Syphilis | -0.457****** | 0.498****** | -0.253 | -0.152 | 0.201 | 0.397****** | -0.139 | 0.033 | 0.169 | -0.022 | -0.013 | 0.395****** | 0.681****** | -0.168 | 0.424****** | 0.663****** | -0.331***** |
| Gonorrhoea | -0.173 | 0.180 | -0.133 | -0.148 | 0.068 | 0.490****** | 0.127 | 0.244 | 0.545****** | 0.100 | 0.332***** | 0.309***** | 0.482****** | -0.333***** | 0.190 | 0.461****** | -0.512****** |
| Acquired immune deficiency syndrome | -0.439****** | 0.574****** | -0.109 | -0.160 | 0.333***** | -0.143 | -0.519****** | -0.357***** | -0.231 | -0.313***** | -0.284 | 0.312***** | 0.309***** | 0.038 | 0.359***** | 0.376****** | 0.008 |
| Measles | -0.253 | 0.165 | -0.034 | -0.015 | 0.040 | 0.124 | -0.014 | -0.032 | -0.068 | -0.104 | -0.176 | 0.299***** | 0.458****** | -0.159 | 0.027 | 0.415****** | -0.294***** |
| **Water and food transmitted diseases** |  |  |  |  |  |  |  |  |  |  |  |  |  |  |  |  |  |
| Infectious diarrhea | -0.220 | 0.213 | -0.074 | -0.175 | 0.249 | 0.274 | 0.036 | 0.034 | 0.257 | -0.024 | 0.199 | 0.066 | 0.521****** | -0.313***** | 0.086 | 0.636****** | -0.501****** |
| Hepatitis A | -0.174 | 0.081 | -0.213 | -0.069 | -0.117 | 0.029 | 0.029 | -0.006 | -0.076 | 0.001 | -0.172 | 0.212 | 0.394****** | -0.233 | -0.045 | 0.361***** | -0.218 |
| Hepatitis E | 0.136 | -0.297***** | 0.113 | 0.024 | -0.159 | 0.260 | 0.434****** | 0.411****** | 0.405****** | 0.292***** | 0.213 | -0.092 | 0.541****** | -0.380****** | -0.136 | 0.553****** | -0.557****** |
| Hepatitis B | -0.041 | 0.002 | -0.195 | 0.162 | -0.147 | 0.312***** | 0.195 | 0.393****** | 0.713****** | 0.103 | 0.221 | 0.234 | 0.533****** | -0.543****** | 0.008 | 0.398****** | -0.583****** |
| Bacillary and amoebic dysentery | -0.421****** | 0.422****** | -0.026 | 0.037 | 0.277 | 0.103 | -0.241 | -0.202 | 0.057 | -0.303***** | -0.076 | 0.277 | 0.392****** | -0.399****** | -0.054 | 0.436****** | -0.356***** |
| Hepatitis C | -0.014 | -0.009 | -0.109 | -0.151 | -0.012 | 0.327***** | 0.187 | 0.242 | 0.453****** | 0.184 | 0.272 | 0.208 | 0.382****** | -0.364***** | 0.042 | 0.311***** | -0.442****** |
| Typhoid and paratyphoid | -0.464****** | 0.496****** | -0.069 | -0.059 | 0.305***** | 0.187 | -0.333***** | -0.200 | -0.089 | -0.301***** | -0.053 | 0.474****** | 0.102 | -0.137 | 0.038 | 0.116 | -0.096 |
| Hepatitis (unspecified) | 0.133 | -0.160 | -0.118 | 0.072 | -0.171 | 0.283 | 0.295* | 0.357* | 0.686** | 0.245 | 0.267 | -0.019 | 0.650** | -0.568** | -0.061 | 0.561** | -0.669** |
| AHC | -0.450** | 0.437** | -0.106 | -0.266 | 0.224 | 0.190 | -0.226 | -0.260 | -0.138 | -0.369** | -0.190 | 0.487** | 0.171 | -0.206 | -0.094 | 0.152 | -0.134 |
| **Airborne transmitted diseases** |  |  |  |  |  |  |  |  |  |  |  |  |  |  |  |  |  |
| Influenza | 0.350***** | -0.407****** | 0.208 | -0.006 | -0.078 | 0.163 | 0.497****** | 0.152 | -0.046 | 0.334***** | 0.347***** | -0.366***** | 0.085 | 0.121 | -0.105 | 0.181 | -0.065 |
| Pulmonary tuberculosis | -0.518****** | 0.566****** | -0.118 | -0.085 | 0.327***** | 0.198 | -0.236 | -0.127 | -0.090 | -0.079 | -0.173 | 0.297***** | 0.290***** | 0.251 | 0.676****** | 0.345***** | 0.144 |
| Scarlatina | 0.300***** | -0.343***** | -0.071 | -0.021 | -0.270 | 0.429****** | 0.526****** | 0.491****** | 0.436****** | 0.561****** | 0.357***** | -0.279 | 0.505****** | -0.115 | 0.125 | 0.445****** | -0.363***** |
| Mumps | -0.467****** | 0.581****** | -0.278 | -0.110 | 0.128 | 0.176 | -0.352***** | -0.082 | -0.012 | -0.207 | -0.278 | 0.418****** | 0.607****** | -0.090 | 0.401****** | 0.619****** | -0.189 |
| Pertussis | -0.470****** | 0.393****** | -0.123 | -0.076 | 0.141 | 0.229 | -0.125 | -0.074 | -0.145 | -0.287***** | -0.287***** | 0.426****** | 0.614****** | -0.396****** | -0.106 | 0.574****** | -0.391****** |
| Rubella | -0.137 | 0.061 | -0.195 | -0.130 | -0.153 | 0.041 | 0.008 | 0.055 | -0.302***** | -0.009 | -0.177 | 0.159 | 0.275 | -0.043 | -0.114 | 0.259 | -0.061 |
| **Vector-borne transmitted diseases** |  |  |  |  |  |  |  |  |  |  |  |  |  |  |  |  |  |
| Malaria | -0.054 | 0.129 | -0.063 | -0.112 | -0.056 | 0.243 | 0.036 | 0.097 | 0.255 | 0.040 | 0.196 | 0.087 | 0.301***** | -0.247 | 0.045 | 0.246 | -0.308***** |
| Dengue | -0.432****** | 0.425****** | 0.076 | 0.067 | 0.255 | -0.221 | -0.387****** | -0.444****** | -0.345***** | -0.425****** | -0.396****** | 0.111 | 0.268 | -0.127 | -0.054 | 0.343***** | -0.108 |
|  | 12 | 13 | 0 | 0 | 3 | 5 | 9 | 6 | 8 | 10 | 6 | 10 | 16 | 7 | 4 | 16 | 11 |

Notes: * at p < 0.05, ** at p < 0.01; AP: Atmosphere pressure, kPa; Precipitation, mm; d; RH: Relative humidity, %; Temperature: ^o^C; WS: Wind speed, m s^-1^; AQI: Air quality index; SC: Social consumption, billion yuan; IO: Industrial output value, billion yuan; FV: Freight volume, million ton; IT: Inbound travel, million travelers; APT: Airport passenger throughput, million passengers.

**References**

Hyndman, R.J., Athanasopoulos, G., 2018. Forecasting: principles and practice. OTexts, Melbourne, Australia.

NASA. (2020). NASA prediction of worldwide energy resources. https://power.larc.nasa.gov/

SBS. (2020). Data dissemination. https://tjj.sh.gov.cn/

SMHC. (2020). Information on epidemic situation of infectious diseases. https://wsjkw.sh.gov.cn/yqxx/index.html
